# An Adult Case of Adenovirus-Associated Acute Disseminated Encephalomyelitis

**DOI:** 10.1155/2023/5528198

**Published:** 2023-05-22

**Authors:** Dewayne Campbell, Gordon S. Wong, Hyun Park, Gavin McLeod

**Affiliations:** Department of Internal Medicine, Yale New Haven Health, Greenwich Hospital, Greenwich, CT, USA

## Abstract

Acute disseminated encephalomyelitis (ADEM) is an autoimmune neurological disease that predominately affects pediatric population. Only a single fatal adult case of adenovirus-associated ADEM has previously been published by Qamar et al. in 2021. Here, we present an adult case of adenovirus-associated ADEM, which was diagnosed early in her clinical course. The patient was treated with the prompt initiation of steroids, intravenous immune globulin (IVIG), and plasmapheresis (PLEX), and the patient recovered fully. This case highlights the importance of early accurate diagnosis for other clinicians to treat adenovirus-associated ADEM in a timely fashion to prevent a potentially fatal outcome.

## 1. Introduction

Acute disseminated encephalomyelitis (ADEM) is an autoimmune inflammatory central nervous system disease that mainly affects the pediatric population with an annual incidence of 0.07 to 0.64 per 100,000 children being reported [1]. The exact annual incidence of ADEM in adult is understudied [2]. Xiong et al. reported that the incidence was 0.27/100,000 among those 15–59 years old and 0.55/100,000 among those over 60 years old in the population in Nanchang, China [3]. Given the wide distribution among pediatric incidence, the adult incidence in Nanchang may not be generalizable to other countries.

A preceding infection or illness is observed in 70–80% of cases [4], such as measles, rubella, varicella zoster, influenza, Epstein–Barr virus, herpes simplex virus, enterovirus, coxsackievirus, *Mycoplasma pneumonia*, *Borrelia burgdorferi*, beta-hemolytic streptococcus, and recently COVID-19 [5, 6]. Precursory symptoms of ADEM in adults include fever, malaise, and headache. Symptoms of encephalopathy seen within day 5 of onset include lethargy and coma. Behavioral changes are seen in 20–56% in adults [7]. Focal neurological deficits vary with the area of the brain affected. These kinds of deficits include vision changes, sensory deficits, and loss of motor functions. Some of the more uncommon symptoms include recurrent seizures, stroke-like symptoms, and dystonia [8].

Upon searching on PubMed on January 2023, we found only one reported case of adenovirus-associated ADEM [9]. Qamar et al. reported an adult case of fatal, rapidly progressing obtundation after a mild upper respiratory adenovirus infection in a 27-year-old female. The initial diagnosis of their case was determined to be adenovirus encephalitis, which was treated with IV antibiotics, antiviral, and steroids. However, the patient's clinical status deteriorated, so the family decided to withdraw care on hospital day 10. After autopsy, the final diagnosis was determined to be ADEM [9]. Here, we present another case of adenovirus-associated ADEM; however, the presented patient recovered fully by the prompt initiation of steroids, IVIG, and PLEX. This case highlights the importance of an early accurate diagnosis of ADEM to guide proper management and successful clinical outcome.

## 2. Case Presentation

A 32-year-old female with a medical history of lymphocytic colitis (not on any immunosuppressor/immunomodulator) and/or irritable bowel syndrome presented with 1-2 weeks of upper respiratory symptoms, 2 days of headache, and 1 day of profound weakness and confusion. She had known sick contacts (viral upper respiratory symptoms) from family members 2-3 weeks before experiencing any neurological symptoms.

**Figure 1:**
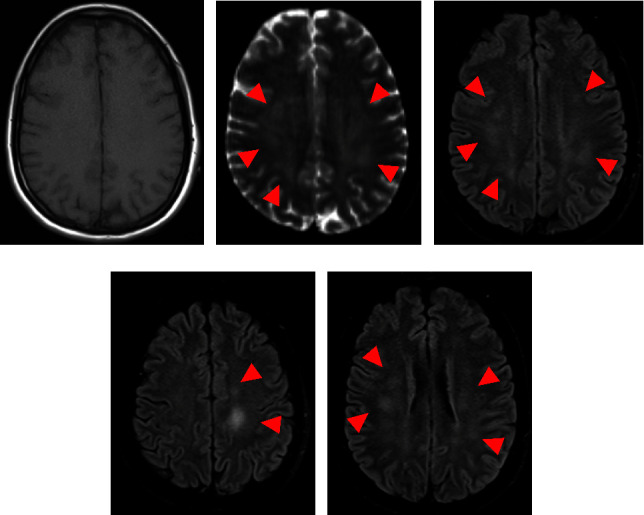
Axial MRI images of the brain at different levels of the brain. (a) T1 image is unremarkable. (b) T2 image and (c) FLAIR image at the same level show multiple foci of abnormal increased signal (red arrow). (d, e) FLAIR images at different levels of the brain demonstrate multiple abnormal foci (red arrow).

In the emergency room, she was awake and conversational but confused. She was observed to have a transient episode of twitching in her right face and arm. Neurological examination revealed no focal neurological deficits and no nuchal rigidity. Vital signs were temperature of 103°F; blood pressure of 100/70 mmHg; heart rate of 116 beats/min; and oxygen saturation of 96% on room air. Blood chemistry revealed normal glucose, electrolytes, and renal and hepatic functions. Complete blood count demonstrated a white blood cell (WBC) count of 9.3 × 1000/*μ*L (65% neutrophils, 24% lymphocytes, and 9% monocytes). Toxicology screen was unremarkable. Nares polymerase chain reaction (PCR) was positive for adenovirus. No specific serotype of adenovirus was tested. Given the concern for encephalitis, a lumbar puncture (LP) was performed. Cerebrospinal fluid (CSF) showed glucose within normal limits, an elevated total protein count of 126 mg/dL, and elevated WBC counts of 67 cells/*μ*L with a lymphocytic predominance of 68%. Immediate CSF PCR was negative for adenovirus, West Nile virus, herpes simplex virus, varicella zoster virus, enterovirus, and parechovirus. A computerized tomography (CT) scan without contrast of the head demonstrated no acute intracranial processes. However, a magnetic resonance imaging (MRI) of the brain with and without intravenous (IV) contrast showed multiple foci of abnormal increased T2-weighted fluid-attenuated inversion recovery (FLAIR) signal in the white matter in the supratentorial brain (Figures 1(a)–1(e)).

She was admitted to the hospital under the medicine service for further investigation and treatment of encephalopathy. Due to the possible bacterial cause of encephalopathy, IV ceftriaxone (2 g every 12 hours) and vancomycin (1.25 g every 12 hours) were started empirically. On the same day of admission, the patient's mental status deteriorated further—she was not following commands with worsening lethargy. The patient was upgraded to the intensive care unit (ICU) for closer neurologic monitoring. The patient was observed to have mouth twitching and tonic-clonic movements of her right upper extremity. EEG showed non-convulsive status epilepticus. She was given levetiracetam and lorazepam; however, due to increased seizure activity, phenytoin and lacosamide were also given. Due to possible autoimmune cause of encephalopathy, methylprednisolone (1000 mg daily) and IVIG (0.4 mg/kg daily) were then started. Acyclovir was also added for potential viral coverage. On day 2 of admission, the patient's oxygen saturation dropped to low 80% on room air and the patient became increasingly lethargic; therefore, she was intubated immediately for airway protection. Pre-intubation chest X-ray (CXR) or arterial blood gas was not performed due to the concern of airway compromise, but post-intubation CXR showed clear lungs and heart size. She was transferred to a tertiary care centre for a higher level of care on the same day.

At the tertiary centre, further diagnostic studies were performed. CSF studies were negative for herpes simplex virus, varicella zoster virus, enterovirus, parechovirus, adenovirus, Eastern equine encephalitis virus, Powassan virus, and *Cryptococcus*. Serum studies were negative for Lyme antibody (Ab), neuromyelitis optica (NMO) Ab, and oligodendrocyte glycoprotein (MOG) Ab. Autoimmune encephalopathy panel was only positive for glutamic acid decarboxylase (GAD) but at a low level at 0.1 nmol/L, which was determined to have unclear significance in this case. Blood sample was not tested for adenovirus as the patient was neither immunocompromised nor immunosuppressed. Given the patient's multifocal neurological deficits, with the presence of encephalopathy and MRI findings of multiple foci of abnormal increased T2/FLAIR signal in the white matter, a diagnosis of adenovirus-associated ADEM was determined after other infectious, neoplastic, vascular, and metabolic causes had been ruled out. She completed a course of IVIG (2 g/kg divided over 5 days), 5 days of plasmapheresis (PLEX), and 5 days of methylprednisolone (1000 mg daily), followed by a prolonged oral steroid taper. Over her 15 days of hospitalization, her mental status ultimately improved, and seizures resolved. She was discharged to a rehabilitation facility with a seizure regimen of only lacosamide. She showed no relapse of ADEM at least 3 months after discharge from the hospital.

## 3. Discussion

ADEM is a rare immune mediated demyelinating disorder of the CNS that is most widely thought to be a post-infectious and post-vaccination autoimmune phenomenon [10]. The etiology of ADEM is still poorly understood, but there are two proposed mechanisms [11]. The first theory is molecular mimicry. When a viral infection occurs, there is a cross-reactive autoimmune reaction against the CNS tissue expressing a protein that mimicked the viral pathogen. The second hypothesis is the post-infectious theory. A neurotropic virus may disrupt the blood-brain barrier (BBB), causing a leakage of CNS-confined auto-antigens, that leads to breakdown of tolerance. Subsequently, immune cells enter via the damaged BBB and attack against CNS proteins [4].

Based on the International Pediatric Multiple Sclerosis Study Group's criteria (IPMSSG's criteria), ADEM is characterized by polyfocal neurological deficits with the presence of encephalopathy and specific MRI and laboratory findings, after other mimics (infectious, neoplastic, vascular, or metabolic) had been ruled out [5]. Neurological deficits typically start within 2–21 days after an infection or vaccination. The most reported neurological deficit is weakness of lower and/or upper extremities (17–77%). Other symptoms or signs include ataxia (10–52%), cranial nerve palsies (11–48%), seizures (4–48%), fever (27–63%), headache and/or vomiting, and meningeal sign (13–43%) [2]. Brain MRI shows hyperintensity in T2-weighted FLAIR images. Lesions are bilateral, asymmetrical, large (>2 cm), and poorly demarcated. They typically occur in deep and subcortical white matter while sparing periventricular white matter, unlike multiple sclerosis [5]. T1-weighted images with gadolinium enhancement are reported in up to 30% of cases [4], indicating active inflammation and loss of BBB integrity [5]. In addition to the brain, spinal cord involvement is reported in up to 33% of patients. T2 hyperintense confluent lesions extending over multiple segments and cord swelling could be seen on spinal MRI [8, 12]. Other laboratory findings including leukocytosis, elevated CRP/ESR, CSF pleocytosis, and elevated CSF protein can be seen [13]. Recently, the measurement of MOG-Ab autoantibodies that are directed against part of the myelin sheath is increasingly utilized. Data suggest that individuals with seropositivity to MOG-Ab are at risk for relapses in both pediatric and adult populations [14].

Once the diagnosis of ADEM has been made, it is crucial to implement therapies to optimize neurocognitive outcomes and prevent fatality, as highlighted in our case. About 50–80% of ADEM is responsive to high-dose IV corticosteroids (level IV evidence of Oxford Centre for Evidence-Based Medicine), which will decrease the inflammatory cytokine cascade, inhibit T cell activation, decrease extravasation of immune cells into CNS, and facilitate apoptosis of activated immune cells [5, 15, 16]. However, when symptoms are severe, including refractory seizures and worsening neurological deficits, as shown in our case, it is vital to initiate either PLEX (level II evidence) and/or IVIG (level IV evidence) [15]. PLEX helps to removes pathologic substances such as circulating pathogenic immunoglobulins, immune complexes, and cytokines [17]. Weinshenker et al. conducted a randomized trial that included 12 patients with multiple sclerosis, 4 patients with transverse myelitis, 1 patient with ADEM, 2 patients with neuromyelitis optica, and 4 patients with other CNS demyelinating disease who have failed to improve after at least 5 days of high-dose parenteral steroids. Treated patient with PLEX showed a 42.1% response rate vs. a 5.9% response rate in controls [18]. The limitation of this study is the low number of patients diagnosed with ADEM, but experts generalized the above result to other CNS demyelinating diseases listed above. Furthermore, IVIG helps to decrease endogenous immunoglobulin production, facilitate degradation of native immunoglobulins, and interfere with pathogenic immune complexes [5]. A review of 20 pediatric and 8 adult cases of monophasic ADEM showed that 70% of pediatric and 50% of adult patients have shown complete recovery after IVIG or IVIG plus steroid [19]. Cyclophosphamide has been documented in the literature as a treatment for patients who only respond partially and very slowly to conventional treatment. Ayed et al. reported a case of dramatic and quick improvement with just 1 dose of cyclophosphamide after patient failed to improve on corticosteroid, IVIG, and PLEX [20]. The systemic antiviral use of cidofovir and probenecid may not be an ideal treatment regime for ADEM as they directly affect viral infection rather than treating the sequela of autoimmunity [21].

The disease course is typically more severe in adult than in pediatric patients. In adults, the duration of hospitalization was longer, and the ICU admission was more frequently required [22]. Ketelslegers et al.'s retrospective study found that the mortality rate of adult is higher than pediatric patients—3 out of 25 adults (12% died) vs. 1 out of 92 children (1%) died [22]. The overall prognosis in terms of functional outcome was lower in adult than in children (children, 100%; young adults, 66.7%; and elderly adults, 75%), which may be related to the reduced plasticity of the adult brain [12, 23]. Furthermore, Koelman et al. found that patient who required therapeutic treatment with IVIG and PLEX had a significantly lower chance of a favorable clinical outcomes (66% vs. 85%) [13]. Although ADEM was once thought to be a monophasic disease, recurrent ADEM has been reported in up to 27% of patients [15]. Therefore, patients with high MOG-Ab level should be closely monitored with repeat brain imaging every 6–12 months.

Here, we presented a second case of adult adenovirus ADEM described in the literature. Although adenovirus in the respiratory sample of this patient could be a chance finding, we believe that the adenovirus was the true culprit. Our patient's polyfocal neurological deficits with encephalopathy within 1-2 weeks of adenovirus upper respiratory infection, multiple foci of abnormal increased T2/FLAIR signal in the white matter, elevated CSF protein, and prompt improvement after steroid, IVIG, and PLEX qualified for ADEM based on the IPMSSG's criteria. In addition, other mimics (infectious, neoplastic, vascular, or metabolic) have been sufficiently ruled out, making a stronger case that adenovirus is the true culprit, causing ADEM in our patient.

## 4. Conclusion

ADEM is an autoimmune neurological disease that predominately affects pediatric population. Here, we presented a second case of adult adenovirus-associated ADEM described in the literature. ADEM is characterized by polyfocal neurological deficits with the presence of encephalopathy and specific MRI and laboratory findings, after other mimics (infectious, neoplastic, vascular, or metabolic) had been ruled out. The disease course and prognosis are typically more severe in adult than in pediatric patients. However, this case highlights the importance of accurate early diagnosis and treatment of adenovirus-associated ADEM to prevent a potentially fatal clinical outcome.

## Data Availability

The health record data used to support the findings of this case report are restricted in order to protect patient privacy. The data used in the discussion were found in peer-reviewed journals and previously published case reports. Appropriate citations and references are included within the article.
